# QTL identification for yield components using single segment substitution lines dissected by rice CSSL-Z799

**DOI:** 10.3389/fpls.2026.1794948

**Published:** 2026-03-12

**Authors:** Xiaodong Wang, Aoni Xiang, Xinyu Fan, Guofeng Li, Minghui Zhao, Dachuan Wang, Kaiyue Yang, Yinghua Ling, Zhenglin Yang, Fangming Zhao

**Affiliations:** Rice Research Institute, Southwest University/Academy of Agricultural Sciences, Southwest University/Chongqing Key Laboratory of Crop Molecular Improvement, Chongqing, China

**Keywords:** additive effects, chromosome segment substitution line (CSSL), quantitative trait locus/loci (QTL) mapping, rice, yield-related traits

## Abstract

As one of the world’s most important cereals, rice (*Oryza sativa* L.) demands sustained yield improvement. However, this goal is challenging because yield components are complex quantitative traits governed by numerous minor-effect genes. To reveal this genetic complexity, Single Segment Substitution Lines (SSSLs) provide an ideal platform for gene identification and designed breeding. Here, we report on a Chromosome Segment Substitution Line (CSSL), Z799, containing 10 substitution segments from the restorer line R225 in the genetic background of Nipponbare. These substitution located on eight different chromosomes, with an average substitution length of 3.0 Mb. Z799 exhibited a complex yield-related phenotype relative to Nipponbare, with several traits being significantly altered. Genetic mapping in a secondary F_2_ population of Nipponbare/Z799 uncovered 27 QTL, but a more efficient SSSL-based approach, which yielded five lines (S1-S5), detected a total of 35 QTL. All five SSSLs significantly enhanced grain length through distinct QTL (*qGL1*, *qGL3*, *qGL12-1*, *qGL12-2*, *qGL12-3*) without compromising grain width. Mechanistically, microscopic observation of lemma cells revealed two divergent pathways: four QTL (*qGL1*, *qGL3*, *qGL12-1*, *qGL12-2*) increased grain length by promoting cell division, whereas *qGL12–3* achieved the same effect by stimulating cell expansion. Our study thus not only identifies critical QTL for yield components but also elucidates their underlying cellular mechanisms, offering a platform for future gene cloning and designed breeding strategies.

## 1 Introduction

Rice (*Oryza sativa* L.), one of the world’s most important food crops, also play a vital role in China’s agricultural production (Gross and Zhao, 2014). Enhancing the yield is still a central objective of ongoing breeding efforts. Rice yield is primarily determined by grain weight, number of grains per panicle, and number of effective panicles (Song et al., 2022). These traits often exhibit complex trade-offs and constraints; deciphering their intrinsic genetic regulatory networks is key to achieving breakthroughs in breeding. Dissecting these quantitative trait loci (QTL) that regulate rice yield traits into specific chromosome segment substitution lines (CSSLs) is therefore essential for achieving high-yield design breeding.

CSSLs enable the precise identification of QTL (Koumproglou et al., 2002; Subudhi et al., 2015) and are also important resources for studying gene function and designed breeding (Zhang et al., 2021). When a CSSL contains only a single substituted segment, it is referred to as a single-segment substitution line (SSSL), which represents a more efficient genetic and breeding resource Zhang (2021). To date, numerous yield-related QTL have been map-based cloned and functionally characterized using CSSLs or near-isogenic lines (NILs). The *GW2* gene encodes a RING-type E3 ubiquitin ligase. It functions by specifically recognizing and degrading positive regulators of the cell cycle, such as *OsSPL16*, through the ubiquitin-proteasome system (UPS). The process suppresses the mitotic activity of lemma epidermal cells and ultimately results in a significant reduction in rice grain width (Song et al., 2007). *GS5* encodes a serine carboxypeptidase and positively regulates grain width, grain filling, and thousand-grain weight in rice (Li et al., 2011; Liu et al., 2017). The *TGW2* gene encodes an F-box-type cell proliferation regulator, OsCNR1, which specifically interacts with the cell cycle inhibitor KRP1 (Kip-related protein 1) to mediate its ubiquitination and degradation. Impairment of this regulatory module reduces cell proliferation activity, ultimately leading to decrease the grain width and thousand-grain weight by approximately 15% and 20%, respectively (Ruan et al., 2020). *GSW3.1* positively regulates grain size and thousand-grain weight (Long et al., 2024). Promoter variation of *SGW5* drives its high expression in wide-grain varieties, positively regulating grain width by influencing lemma cell size and division (Abbas et al., 2024). *GS3* is a major QTL controlling grain size, functioning as a negative regulator in modulating grain and organ size (Mao et al., 2010; Zhang et al., 2012). *GL7* is a QTL on chromosome 7 that primarily controls grain length and width in rice (Wang et al., 2015). It encodes a cell elongation regulator functionally homologous to the Arabidopsis LONGIFOLIA protein. In large-grain germplasm, a 17.1-kb tandem repeat sequence upstream of the *GL7* gene enhances grain length and significantly improves rice appearance quality (Lee et al., 2006; Wang et al., 2015). *OsGSW3.2* (also known as *qGL3.5*) is a natural variant allele identified on chromosome 3, derived from the *Oryza rufipogon* inbred line Huaye3 (HY3). It negatively affects grain size by regulating cell proliferation and the brassinosteroid (BR) signaling pathway, and its natural variation is closely associated with the differentiation between *indica* and *japonica* rice subspecies (Chu et al., 2006). OsSPL16/*GW8* is a SBP domain transcription factor. It directly binds to the promoter region of the *GW7* gene and inhibits its transcriptional activity. This regulatory mechanism constitutes a network for the negative control of grain width in rice (Wang et al., 2015). *GS9* encodes a transcriptional regulatory protein that lacks clearly defined conserved domains and functions to regulate grain shape by modulating cell division patterns (Zhao et al., 2018). *OsSPL13* (encoded by *GLW7*) is a rice-specific transcription factor that positively regulates lemma cell size, significantly promoting longitudinal grain elongation and grain weight (Sheng et al., 2022). As a newly identified positive regulator, *GW5* mediates the brassinosteroid signaling pathway, leading to increase rice grain width and weight (Liu et al., 2017). *Gn1a* regulates rice grain number per panicle by encoding *OsCKX2*; a cytokinin oxidase/dehydrogenase; its downregulation elevates cytokinin levels in the panicles, resulting in more primary branches and enhanced yield (Kurakawa et al., 2007). *GRAIN SIZE AND NUMBER1 (GSN1)* encodes a mitogen-activated protein kinase phosphatase, OsMKP1. Within the rice cell signaling network, OsMKP1 specifically binds to and dephosphorylates OsMPK6, inactivating this kinase. The action negatively regulates the OsMKKK10-OsMKK4-OsMPK6 cascade, ultimately influencing the number of secondary branches and grain size (Duan et al., 2014; Guo et al., 2018; Liu et al., 2015). The panicle architecture gene *DEP1* is directly activated by the transcription factor *IPA1* (*OsSPL14*). *IPA1* expression is negatively regulated by *OsmiR156*; a point mutation that escapes this repression causes IPA1 accumulation, which in turn promotes primary branching and suppresses tillering by activating target genes including *DEP1*, thereby shaping ideal panicle structure (Jiao et al., 2010; Miura et al., 2010). The cloning of these QTL has laid a solid foundation for molecular design breeding of high-yield rice.

Although some yield-related QTL have been cloned, many minor-effect QTL remain unidentified. To address this, it is essential to develop a single-segment substitution lines (SSSLs) library to eliminate background interference. However, SSSL development is a challenging systematic project, requiring gradual screening to reduce multiple substitution segments to a single segment. Based on previous research using the rice chromosome segment substitution line Z255―which carries 18 substitution segments in Nipponbare genetic background (Zhang et al., 2025)―this study further utilized the CSSL-Z799 (derived from Nipponbare/Z255 and containing 10 substitution segments) as research material. We performed QTL mapping for yield-related traits and developed secondary single-segment substitution lines. Using five derived SSSLs, additive effect analysis of QTL for yield traits were analyzed, and cytological examinations of grains was conducted for SSSLs harboring grain shape QTL. This work lays the foundation for the future map-based cloning of related genes, while also provides reliable genetic information and resources for molecular design breeding in rice.

## 2 Materials and methods

### 2.1 Plant materials

Rice CSSL-Z799 was developed through marker-assisted selection (MAS) from the progeny (F_2:5_) of a cross between Nipponbare and Z255―a CSSL carrying 18 substitution segments from the *indica* restorer line R225 in the Nipponbare genetic background. The resulting CSSL contains 10 substitution segments (Zhang et al., 2025). The recipient parent, Nipponbare, is a high-quality *japonica* cultivar with a fully sequenced genome. While the donor parent, R225, is an elite *indica* restorer line developed by the Rice Research Institute.

Based on the MAS-derived background of Z799, this study used 16 polymorphic molecular markers (RM8111, RM7202, RM1268, RM5928, RM6303, RM172, RM8243, RM7027, RM8206, RM474, RM3590, RM6404, RM1246, RM27819, RM28258, RM519) corresponding to the 10 substitution segments and 32 polymorphic SSR markers outside these segments to verify the substitution segments and assess genetic background purity using 10 Z799 plants. Banding patterns identical to the recipient parent Nipponbare were scored as “-1”; those matching the donor parent R225 were scored as “1”; and heterozygous banding patterns were scored as “0”.

A secondary F_2_ population comprising 200 individuals, derived from a cross between Nipponbare and Z799, was used for preliminary QTL mapping. Based on the phenotypic evaluations and the preliminary QTL results, secondary segment substitution lines were subsequently developed in the following year.

### 2.2 Plant materials and cultivation

#### 2.2.1 Hybridization stage (2021)

In July 2021, crosses between the recipient parent Nipponbare and Z799 were conducted at the Xiema Experimental Base of Southwest University to generate hybrid seeds. In September of the same year, these F_1_ hybrid seeds were planted at the Hainan Base to produce seeds for the F_2_ population.

#### 2.2.2 Field experiments (2022-2024)

2022 Cycle: On March 10, seeds of the parental lines (Nipponbare, R225, Z799) and the F_2_ population were sown. On April 18, thirty seedlings per parental line and 200 F_2_ plants were transplanted using the spacing of 16.5 cm inter-row × 26.4 cm intra-row, with 10 plants per row.

2023 Cycle: On March 10, the seeds of parental lines and five F_2_-derived SSSLs candidates were sown. On April 18, thirty plants per line were transplanted using identical spacing.

2024 Cycle: On March 12, the seeds of parental lines and the developed five SSSLs (S1-S5) were sown. On April 20, thirty plants per line were transplanted with identical spacing.

All trials were managed following standard Chongqing regional agronomic practices to ensure experimental consistency across generations.

### 2.3 Evaluation of yield-related traits

At full maturity, 10 individuals were randomly sampled from the plots of Nipponbare, Z799 and SSSLs (S1-S5) respectively, along with 200 individual plants from the F_2_ population. Fourteen yield-related traits were investigated for the parental lines, F_2_ population according to the method described by Ma et al. (2019) and Wang et al. (2021). Plant height was measured as the distance from the top of the highest panicle to the field surface in meters for each plant. Panicle numbers, panicle length, number of primary branches, number of secondary branches, number of spikelets per panicle, number of grains per panicle, yield per plant were measured using all the effective panicles in the plant. The total length and width of 10 grains lined up were measured with a 20-cm ruler for three replications then used to calculate average per-grain values of each plant. Ratio of grain length-to-width was calculated as grain length divided by grain width. The 1000-grain weight of Nipponbare and Z799 was measured from random samples of 3000 grains, from which 1000-grain subsets were weighed on an electronic balance, with three repetitions. The 1000-grain weight of each F_2_ plant was determined as the weight of 200 grains, multiplied by 5, with three repetitions. The seed-setting rate was calculated as grains per panicle as a percentage of the number of spikelets per panicle. The seed setting density was determined as spikelet number per 10 cm of panicle length. The mean value, standard deviation, and relevant statistical analyses for each trait were calculated using Microsoft Excel 2016.

### 2.4 QTL mapping

Four weeks after transplanting, leaf samples were collected from individual F_2_ plants, Z799, Nipponbare, and secondary segment substitution lines. Genomic DNA was extracted via the CTAB method and used as a template for PCR amplification. Sixteen polymorphic SSR markers corresponding to the 10 substitution segments in Z799 were used as primers. PCR was performed in a 12.5 μL reaction mixture containing 1.25 μL of 10× PCR buffer, 0.65 μL of 25 mmol L^−1^ MgCl_2_, 0.5 μL of 2.5 mmol L^−1^ dNTPs, 8.0 μL of ddH_2_O, 1.0 μL of 10 μmol L^−1^ primers, 1.0 μL of template DNA, and 0.1 μL of 5 U μL^−1^ Taq DNA polymerase. The PCR protocol consisted of initial denaturation at 94 °C for 3 min, followed by 35 cycles of denaturation at 94 °C for 20 s, annealing at 56 °C for 20 s, and extension at 72 °C for 40 s, with a final extension at 72 °C for 7 min. The PCR products were separated by 10% native polyacrylamide gel electrophoresis (PAGE) and visualized by rapid silver staining. Banding patterns were scored as “1” for Z799 type, “-1” for Nipponbare type, “0” for heterozygous, and “.” for missing data. Based on phenotypic data from the 200 individual plants, QTL analysis was performed using the Mixed Linear Model (MLM) in SAS 9.3 (SAS Institute Inc., Cary, NC, USA). A significance threshold of *P < 0.05* was applied for declaring the presence of a quantitative trait locus (QTL).

### 2.5 Development of single segment substitution lines

Based on the QTL mapping results, five F_3_ plants harboring only a single homozygous substituted segment and 0–1 heterozygous markers were selected from the F_2_ population using marker-assisted selection (MAS). These were then grown as individual lines. From each line, leaves of 20 plants were sampled for DNA extraction, and genotyping was performed using markers for the substitution segments and heterozygous markers. The scoring criteria were as follows: target substitution segment markers were scored as “1” (identical to Z799), and heterozygous markers were selected as “0” (identical to Nipponbare).

### 2.6 QTL identification and additive effect analysis in single segment substitution Lines

Since each SSSL differs from Nipponbare only in a single chromosomal segment, phenotypic differences can be attributed to the unique substitution segment. The genetic model was defined as follows: for Nipponbare, *P_0_ = μ_0_+ϵ* (where *μ_0_* is the mean and *ϵ* is the error); for an SSSL, *Pi = μ_0_ + a_i_ + ϵ* (where *a_i_* is the additive effect). Thus, QTL for yield traits in each SSSL were identified using a *t*-test: the null hypothesis (*H_0_*) stated that no QTL controlling the trait was present in the substituted segment of SSSLi, while the alternative hypothesis (*H_1_*) stated that a QTL was present. When *P < 0.05*, *H_1_* was accepted, indicating the presence of a QTL for a specific trait in SSSLi. The additive effect *a_i_* was estimated as half the difference between *p_i_* and *p_0_* (Li et al., 2022).

### 2.7 Cytological analysis of glume using scanning electron microscopy in SSSLs and Nipponbare

At the booting stage completion and prior to heading, 3 grains from the middle part of the main panicle of each plant were taken from the 3 plants per SSSL and Nipponbare. The inner and outer epidermal cells of the glume in Nipponbare and the five SSSLs (S1–S5) were examined using a Hitachi SU3500 scanning electron microscope (Hitachi, Tokyo, Japan) equipped with a freezing stage (–40 °C) under low-vacuum conditions.

## 3 Results and analysis

### 3.1 Identification of chromosomal substitution segments in Z799

**Figure 1:**
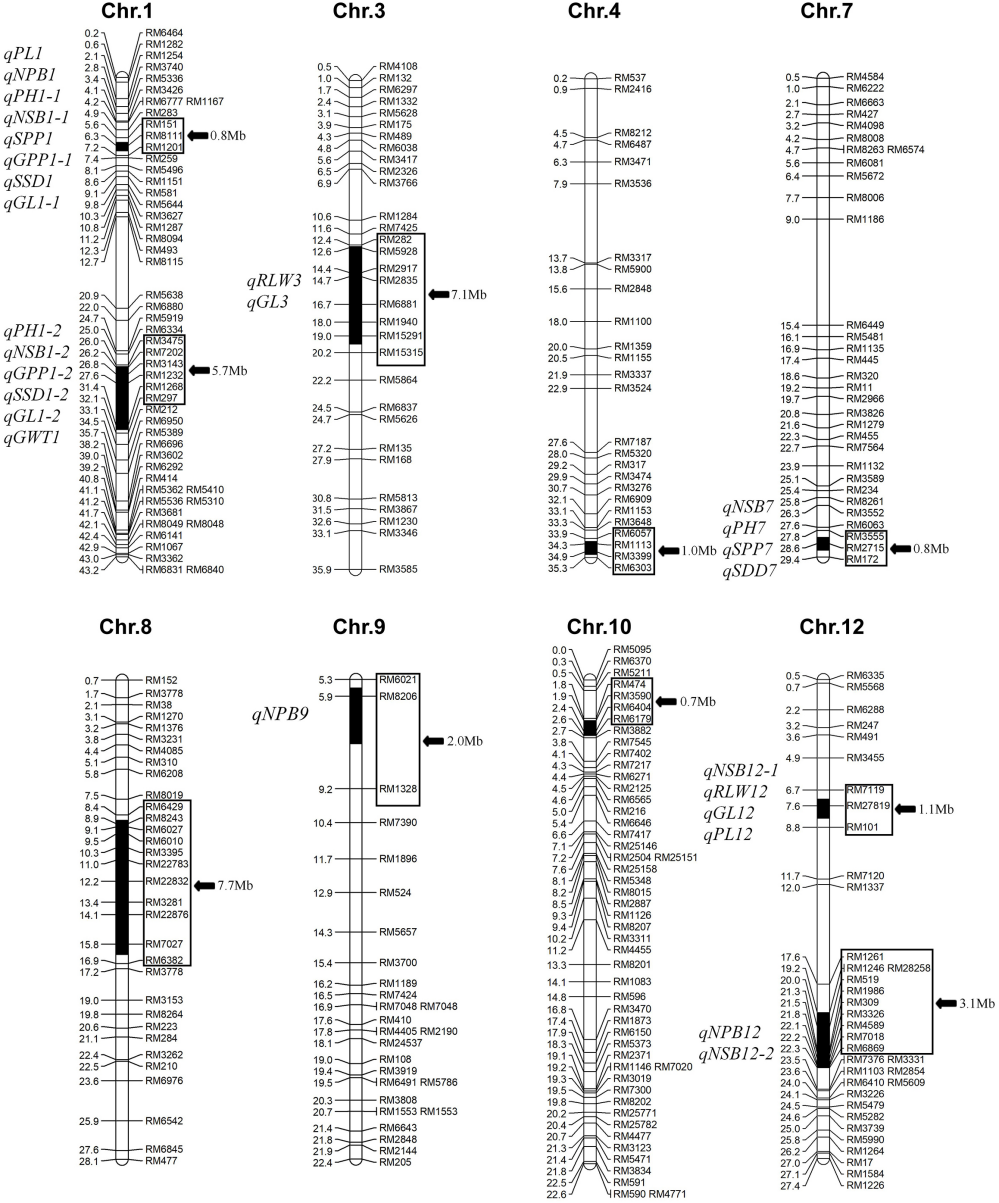
Substitution segments in Z799 and detected QTL. The physical distance (Mb) and mapped QTL were marked at the left of each chromosome, and marker name with substitution lengths (indicated by black arrows) were on the right. Black regions represent the substitution fragment, while white region represents the genetic background of Nipponbare. Chromosomes not shown are identical to Nipponbare. Trait abbreviations in QTL names are as follows: PH, plant height; GL, grain length; NSB, number of secondary branches; NPB, number of primary branches; RLW, ratio of length to width; GWT, 1000- grain weight; GW, grain width; PL, panicle length; GPP, grain number per panicle; SPP: spikelet number per panicle; SSR, seed setting ratio; SSD, seed setting density.

Analysis of substitution segments and genetic background purity in 10 Z799 plants confirmed the accuracy of all 10 target segments and the absence of residual segments from donor line R225. The 10 substitution segments in Z799 were located on chromosomes 1, 3, 4, 7, 8, 9, 10, and 12, with a total length of was 30 Mb. The individual segments range from 0.8 Mb to 7.7 Mb in length, averaging 3.0 Mb (**Figure 1**).

### 3.2 Yield-related traits analysis of Z799 and Nipponbare

**Figure 2:**
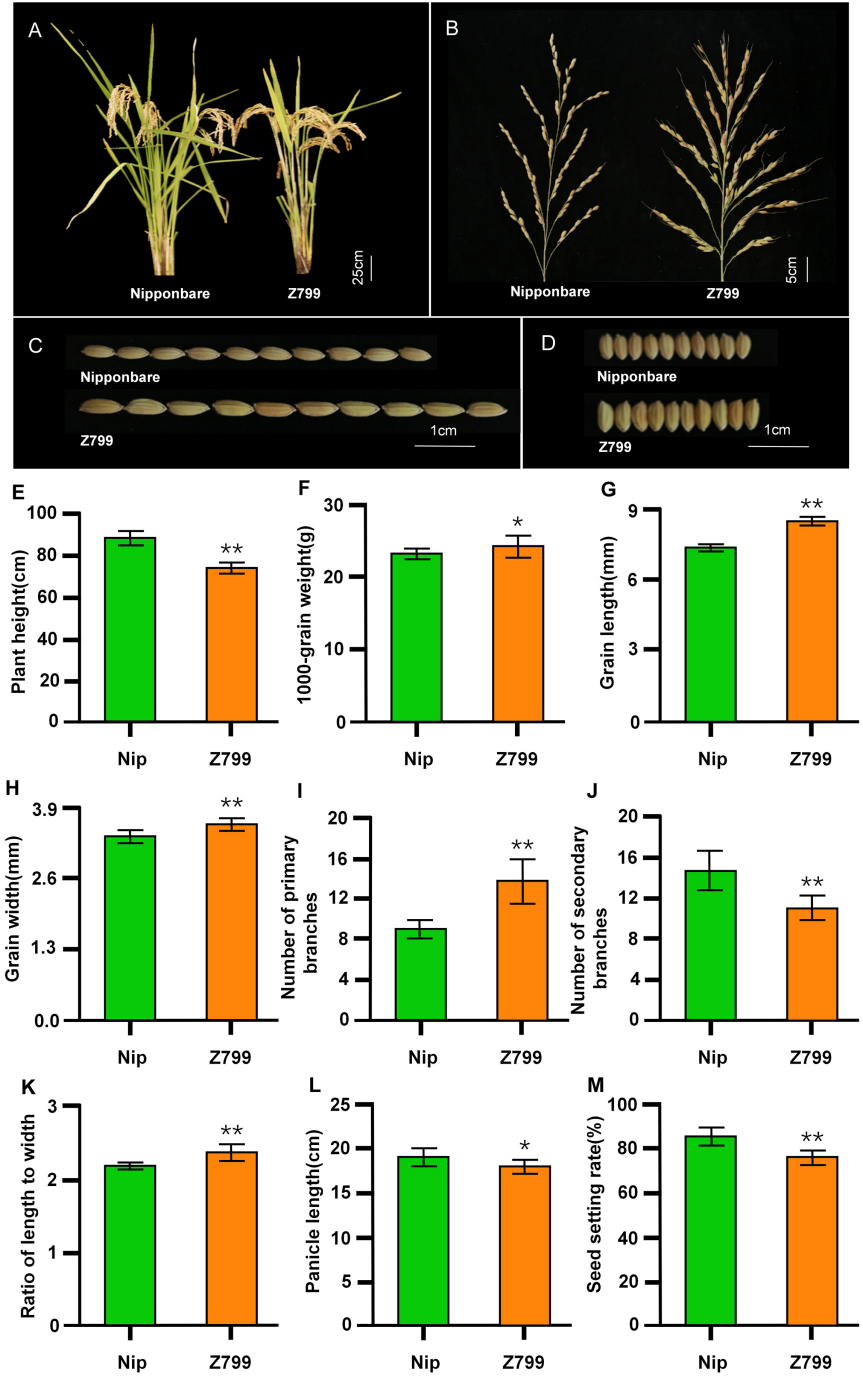
Analysis of yield-related traits in Nipponbare and Z799**. (A–D)** Plant type **(A)**, panicle type **(B)**, grain length **(C)** and grain width **(D)** of Nipponbare and Z799. **(E–M)** Statistical analysis of 9 traits between Nipponbare and Z799. **(E)** Plant height, **(F)** 1000-grain weight, **(G)** Grain length, **(H)** Grain width, **(I)** Number of primary branches number, **(J)** Number of secondary branches, **(K)** Ratio of length to width, **(L)** Panicle length, **(M)** Seed setting ratio. * and ** indicate significant differences between Nipponbare(Nip) and Z799 at the 0.05 and 0.01 levels using two-tail t-test, respectively.

Compared to Nipponbare, the plant height (73.71 cm) of Z799 was significantly reduced by 16.34% (Nipponbare: 88.11 cm) (**Figures 2A, E**). The panicle length (18.00 cm) of Z799 was 5.61% shorter than that (19.07 cm) of Nipponbare (**Figures 2B, L**). In contrast, the grain length (8.72 mm) and grain width (3.45 mm) of Z799 were significantly increased by 16.89% and 0.58%, respectively, relative to Nipponbare (7.46 mm and 3.43 mm) (**Figures 2C, D, G, H**). The number of secondary branches (11.00) of Z799 was significantly decreased by 24.35% compared to that of Nipponbare (14.54) (**Figure 2J**); whereas the seed setting rate (91.76%) of Z799 was significantly increased by 12.37% relative to that of Nipponbare (81.66%) (**Figure 2M**).Conversely, the number of primary branches (13.88) and the length-to-width ratio (2.37) of Z799 were significantly higher than those of Nipponbare (9.23 and 2.17, respectively) (**Figures 2I, K**), corresponding to increases of 50.38% and 9.22%, respectively. Furthermore, 1000-grain weight of Z799 (24.41 g) was 5.30% greater than that (23.18 g) of Nipponbare (**Figure 2F**).

### 3.3 QTL mapping for yield-related traits carried by substitution segments in Z799

Twenty-seven QTL controlling yield-related traits were identified using the secondary F_2_ population derived from Nipponbare/Z799. These QTL were distributed across five chromosomes: 1, 3, 7, 9, and 12. They comprised three for plant height (PH), two for panicle length(PL), four for the number of primary branches(NPB), four for the number of secondary branches(NSB), two for spikeletes per panicle(SPP), two for grains per panicle(GPP), three for seed-setting density(SSD), four for grain length(GL), two for the ratio of length to width(RLW), and one for 1000-grain weight(GWT). The phenotypic variation explained (PVE) by these QTL ranged from 2.23% to 17.45%, with six QTL (*qPL1*, *qNPB1*, *qNPB7*, *qPH1-2*, *qSPP1*, *qSSD1-1*) each exceeding 10%. On chromosome 1, seven QTL (*qPH1-1*, *qPL1*, *qNPB1*, *qNSB1-1*, *qSPP1*, *qGPP1-1*, *qSSD1-1*, *qGL1-1*) were closely linked to the marker RM8111. Their additive effects from the restorer line R225 were as follows: *qPH1–1* increased plant height by 0.51 cm, *qNPB1–1* increased the number of primary branches by 0.43, *qPL1* increased panicle length by 1.59 cm, *qNSB1–1* increased the number of secondary branches by 1.64, *qSPP1* increased the total number of grains per panicle by 10.70, *qGPP1–1* increased the number of filled grains per panicle by 5.88, and *qGL1–1* increased grain length by 0.05 mm. Another six QTL (*qPH1-2*, *qNSB1-2*, *qGPP1-2*, *qSSD1-2*, *qGL1-2*, and *qGWT1*) were closely linked to the marker RM1268. The additive effects from R225 were: *qPH1–2* decreased plant height by 2.96 cm, *qNSB1–2* decreased the number of secondary branches by 1.37, *qGPP1–2* decreased the number of grains per panicle by 4.63, *qSSD1–2* decreased grains number per 10 cm panicle length by 1.96, *qGL1–2* increased grain length by 0.10 mm, and *qGWT1* increased thousand-grain weight by 0.39 g. On chromosome 3, *qGL3* and *qRLW3* were closely linked to the marker RM5928. The additive effects from R225 were: *qGL3* increased grain length by 0.05 mm, and *qRLW3* increased the length-to-width ratio by 0.02. On chromosome 7, four QTL (*qNPB7*, *qPH7*, *qSPP7*, and *qSSD7*) were closely linked to the marker RM172. The additive effects from R225 were: *qNPB7* increased the number of primary branches by 0.36, *qPH7* increased plant height by 1.60 cm, *qSPP7* increased the spikelets per panicle by 5.34, and *qSSD7* increased grains per 10 cm panicle length by 2.02. On chromosome 12, four QTL (*qPL12*, *qNSB12-1*, *qGL12*, and *qRLW12*) were closely linked to the marker RM27819. The additive effects from R225 were: *qPL12* decreased plant height by 0.47 cm, *qNSB12–1* decreased the number of secondary branches by 1.19, *qGL12* increased grain length by 0.06 mm, and *qRLW12* increased ratio of length-to-width by 0.02.Additionally, two QTL (*qNPB12* and *qNSB12-2*) were linked to the second substitution segment marker RM519, with additive effects from R225 of increasing primary branches by 0.31 and increasing secondary branches by 1.71, respectively (**Table 1**).

**Table 1:** QTLs for related traits identified in the Nipponbare/Z799 secondary F_2_ population.

Traits	QTL	Chromosome Chr.	Linked marker	Additive effect	Expained var.%	*P*-value
Panicle length (cm)	*qPL1*	1	RM8111	0.51	10.61	<0.0001
	*qPL12*	12	RM27819	-0.47	9.62	0.0075
Number of primary branches	*qNPB1*	1	RM8111	0.43	17.45	<0.0001
	*qNPB7*	7	RM172	0.36	12.58	<0.0001
	*qNPB9*	9	RM8206	0.19	3.45	0.0240
	*qNPB12*	12	RM519	0.31	9.48	0.0028
Plant height (cm)	*qPH1-1*	1	RM8111	1.59	3.14	0.0221
	*qPH1-2*	1	RM1268	-2.96	10.96	<0.0001
	*qPH7*	7	RM172	1.60	3.27	0.0194
Number of secondary branches	*qNSB1-1*	1	RM8111	1.64	8.40	0.0004
	*qNSB1-2*	1	RM1268	-1.37	5.86	0.0064
	*qNSB12-1*	12	RM27819	-1.19	4.49	0.024
	*qNSB12-2*	12	RM519	1.71	9.29	0.0021
Spikelets per panicle	*qSPP1*	1	RM8111	10.70	16.54	<0.0001
	*qSPP7*	7	RM172	5.34	4.25	0.0106
Grains per panicle	*qGPP1-1*	1	RM8111	5.88	5.35	0.0018
	*qGPP1-2*	1	RM1268	-4.63	3.34	0.0185
Seed-setting density	*qSSD1-1*	1	RM8111	3.83	13.19	<0.0001
	*qSSD1-2*	1	RM1268	-1.96	3.49	0.0306
	*qSSD7*	7	RM172	2.02	3.76	0.0149
Grain length (mm)	*qGL1-1*	1	RM8111	0.05	2.44	0.0491
	*qGL1-2*	1	RM1268	0.10	9.23	0.0005
	*qGL3*	3	RM5928	0.05	2.66	0.0263
	*qGL12*	12	RM27819	0.06	2.81	0.0351
Ratio of length to width	*qRLW3*	3	RM5928	0.02	3.53	0.0071
	*qRLW12*	12	RM27819	0.02	4.15	0.0129
1000-grain weight (g)	*qGWT1*	1	RM1268	0.39	2.47	0.0270

In addition, we also found that some QTL were always detected in cluster. For example, *qPL1*, *qNPB1*, *qNSB1-1*, *qPH1-1*, *qSPP1*, *qGPP1-1*, *qSSD1–1* and *qGL1* linked to the same marker, so was *qPL12*, *qNSB12–1* and *qGL12*, as well as *qNPB7*, *qPH7*, *qSPP7* and *qSSD7*. *etc*. Whether these traits correlated? We analyzed the Pearson correlation coefficient for 14 yield-related traits in the F_2_ population by IBM SPSS25.0 statistical software (**Table 2**). The 1000-grain weight (GWT) was significantly negatively correlated with panicle number (PN) (r = −0.191) but positively correlated with grain width (GW) (r = 0.283). Yield per plant (YD) showed strong positive correlations with both panicle number (PN) (r = 0.604) and plant height (PH) (r = 0.200). The number of primary branches per panicle (NPB) was positively correlated with panicle length (PL) (r = 0.323), while the number of secondary branches (NSB) was positively correlated with both NPB (r = 0.210) and PL (r = 0.213). Spikelets per panicle (SPP) exhibited strong positive correlations with grains per plant (GPP) (r = 0.764) and NSB (r = 0.217). Seed setting rate (SSR) was significantly positively correlated with GPP (r = 0.580) and SPP (r = 0.453). The ratio of grain length-to-width (RLW) showed a significant positive correlation with grain length (GL) (r = 0.314), but negative correlations with grain width (GW) (r = −0.412) and 1000-grain weight (GWT) (r = −0.193). Seed setting density (SSD) was significantly positively correlated with GPP (r = 0.610), SPP (r = 0.773), and SSR (r = 0.320) (**Table 2**). These correlation patterns suggest pleiotropic effects among certain QTL clusters. Specifically, *qPL1*, *qNPB1*, *qNSB1-1*, *qSPP1*, *qGPP1-1*, and *qSSD1–1* appear to represent a pleiotropic region, whereas *qGL1* and *qPH1–1* should be only loosely associated with this group. Similarly, *qNPB7*, *qPH7*, *qSPP7*, and *qSSD7* likely share pleiotropic effects. Additionally, *qPL12* and *qNSB12–1* appear to be pleiotropic, while *qGL12* should be only weakly linked.

**Table 2:** Pearson correlation coefficient among yield-related traits in the F2 population.

Traits	PH	PN	GL	GW	GWT	YD	PL	NPB	NSB	GPP	SPP	SSR	RLW	SSD
PH	1													
PN	0.121	1												
GL	0.015	-0.122	1											
GW	-0.025	-0.071	0.018	1										
GWT	0.009	-0.191*	0.122	0.283**	1									
YD	0.200*	0.604**	0.081	0.005	0.021	1								
PL	0.129	0.047	0.021	0.038	0.005	0.102	1							
NPB	0.106	0.009	-0.048	0.027	-0.009	0.039	0.323**	1						
NSB	0.190	0.141	-0.012	0.009	-0.031	0.146	0.213*	0.210*	1					
GPP	0.019	-0.025	0.009	-0.042	-0.045	0.027	0.089	0.020	0.070	1				
SPP	0.112	0.043	-0.018	-0.019	-0.034	0.090	0.171	0.122	0.217*	0.764**	1			
SSR	0.020	-0.022	0.032	0.001	-0.012	0.013	0.043	0.007	0.018	0.580**	0.453**	1		
RLW	-0.018	0.027	0.314**	-0.412**	-0.193*	0.025	-0.021	-0.005	-0.042	0.018	0.006	0.024	1	
SSD	0.092	0.011	-0.020	0.002	-0.040	0.066	0.141	0.072	0.166	0.610**	0.773**	0.320**	-0.011	1

*and** indicate the coefficient of correlation between two traits existing significant difference at 0.05 and 0.01 level, respectively. No* indicates no significant difference at 0.05 level.

### 3.4 Development of single segment substitution lines from Z799

**Figure 3:**
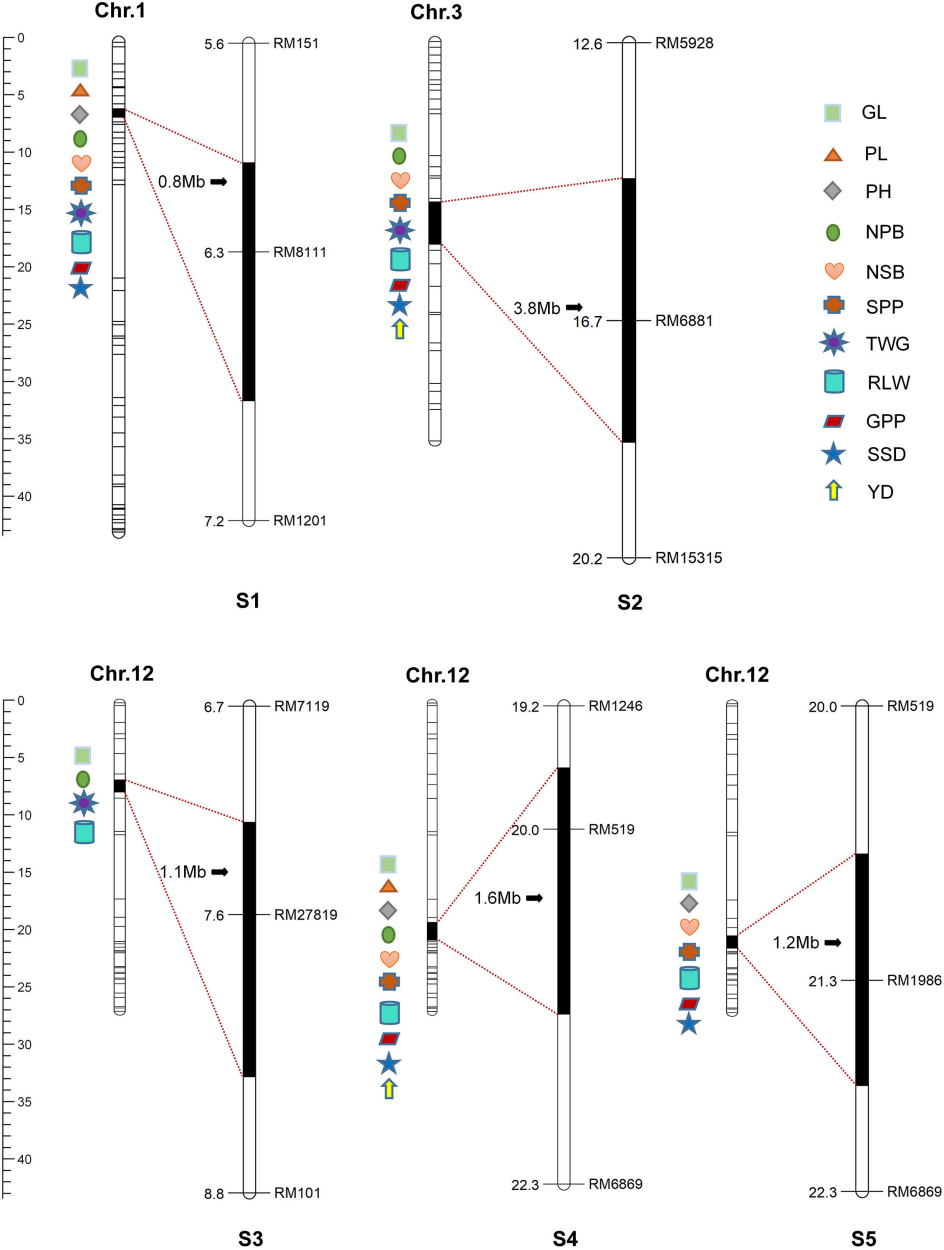
QTL distribution in secondary single segment substitution lines (S1-S5). S1(Chr.1: RM151--RM8111--RM1201); S2(Chr.3: RM5928--RM6881--RM15315); S3(Chr.12: RM7119--RM27819--RM101); S4(Chr.12: RM1246--RM519--RM6869); S5(Chr.12: RM519--RM1986--RM6869). GL, Grain length; PL, Panicle length; PH, Plant height; NPB, Number of primary branches; NSB, Number of secondary branches; SPP, Spikelet number per panicle; GWT, 1000- grain weight; RLW, Ratio of the length to width; GPP, Grain number per panicle; SSD, Seed setting density; SSR, Seed setting rate.

Based on the QTL mapping results, five single segment substitution lines (S1–S5) were. further developed. Among them, S1 and S2 harbored substitution segments on Chromosome 1 and 3, respectively, while S3, S4 and S5 all carried substitution segments on Chromosome 12. Detailed substitution information was provided in **Figure 3**.

### 3.5 Additive effect analysis and detection of QTL for yield traits in developed SSSLs (S1-S5)

**Figure 4:**
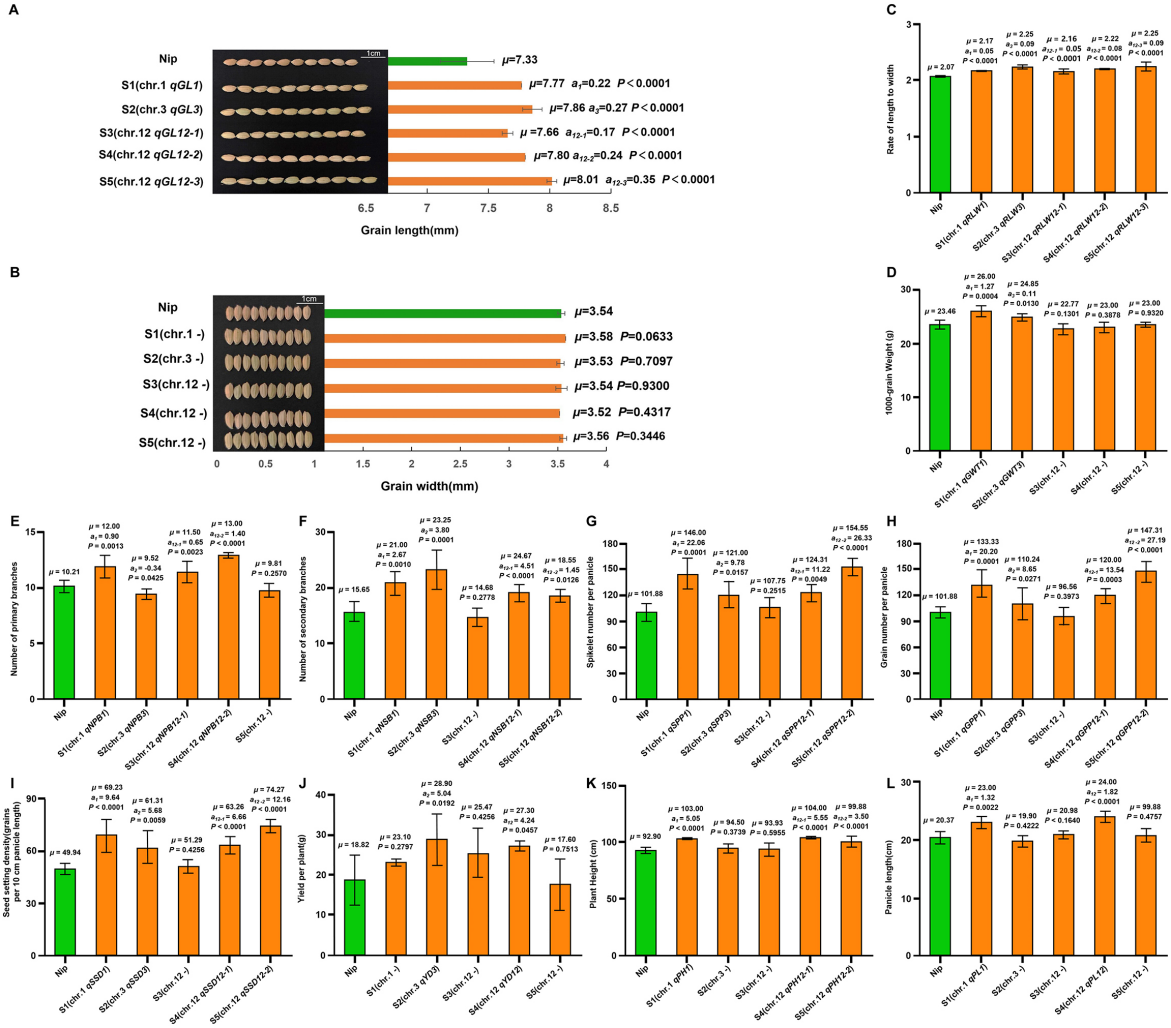
Additive effect analysis of QTL for yield –related traits in S1-S5. **(A–L)** the identification and additive effects of QTL for different yield-related traits carried by SSSLs(S1–S5). **(A)** Grain length; **(B)** Grain width; **(C)** Ratio of length-to-width; **(D)** 1000-grain weight; **(E)** Number of primary branches; **(F)** Number of secondary branches; **(G)** Spikelet number per panicle; **(H)** Grain number per panicle; **(I)** Seed-setting density; **(J)** Yield per plant; **(K)** Plant height; **(L)** Panicle length. The bottom lowercase letters above the column indicate the Duncan’s multiple comparison results (*P* < 0.05). *μ*: Mean trait value of each line, *a_i_*: additive effect of QTL. p < 0.05 in SSSL indicates a significant QTL identified within the substitution segment of the SSSL; *p* > 0.05 indicates no QTL existed in the SSSL_i._ S1(Chr.1: RM151--RM8111--RM1201); S2(Chr.3: RM5928--RM6881--RM15315); S3(Chr.12: RM7119--RM27819--RM101); S4(Chr.12: RM1246--RM519--RM6869); S5(Chr.12: RM519--RM1986--RM6869).

A total of 35 QTL for yield-related traits were identified in the single segment substitution lines S1–S5, including five for grain length, three for spikelets per panicle, three for grains per panicle, five for ratio of length-to-width, three for 1000-grain weight, four for number of primary branches, three for number of secondary branches, three for seed-setting density, two for yield per plant, two for plant height, and two for panicle length. Among them, 15 QTL (*qPL1*, *qNPB1*, *qNPB12-2*, *qPH1*, *qNSB1*, *qNSB12*, *qSPP1*, *qGPP1*, *qSDD1*, *qGL1*, *qGL12-1*, *qGL3*, *qRLW3*, *qRLW12-1*, *qGWT1*) were also detected in the aforementioned F_2_ population, indicating stable inheritance across years. The remaining 18 QTL (*qGL12-2*, *qSPP3*, *qSPP12*, *qGPP3*, *qGPP12*, *qRLW1*, *qRLW12-2*, *qGWT3*, *qGWT12*, *qNPB3*, *qNPB12-1*, *qNSB3*, *qSDD3*, *qSDD12*, *qYD3*, *qYD12*, *qPH12*, *qPL12*) were detected only in the SSSLs (**Figure 3**, **Figures 4A–L**), demonstrating higher QTL detection efficiency of SSSLs compared to the F_2_ population.

The grain length of QTL–carrying SSSLs S1–S5 (*qGL1*, *qGL3*, *qGL12-1*, *qGL12-2*, *qGL12-3*,with additive effects of 0.22, 0.27, 0.17, 0.24 and 0.35 mm, respectively) was significantly greater (7.77, 7.86, 7.66, 7.80, and 8.01 mm) than that (7.33 mm) of Nipponbare (**Figure 4A**). In contrast, grain width did not differ between these SSSLs (3.58, 3.53, 3.54, 3.52 and 3.56 mm), and Nipponbare (3.54 mm) (**Figure 4B**). The ratio of grain length-to-width was significantly higher in S1–S5 (carrying *qRLW1*, *qRLW3*, *qRLW12-1*, *qRLW12-2*, and *qRLW12-3*, with additive effects of 0.05, 0.09, 0.05, 0.08, and 0.09, respectively) compared to Nipponbare (2.17, 2.25, 2.16, 2.22, and 2.25 vs. 2.07; **Figure 4C**).

1000-grain weight was significantly increased in S1 (carrying *qGWT1*, a=1.27 g; 26.00 g) and S2 (carrying *qGWT3*, a=0.11 g; 24.85 g) relative to Nipponbare (23.46 g), whereas S3–S5, which lack 1000-grain weight QTL, showed no significant difference (22.77, 23.00, and 23.00 g; **Figure 4D**). For number of primary branches, S1 (*qNPB1*, a=0.90), S3 (*qNPB12-1*, a=0.65), and S4 (*qNPB12-2*, a=1.40) had significantly more branches (12.00, 11.50, and 13.00) than Nipponbare (10.21), while S2 (*qNPB3*, a=-0.34) had significantly fewer (9.52) and S5 (no QTL) showed no difference (9.81; **Figure 4E**). Number of secondary branches was significantly higher in S1 (*qNSB1*, a=2.67), S2 (*qNSB3*, a=3.80), S4 (*qNSB12-1*, a=4.51), and S5 (*qNSB12-2*, a=1.45) compared to Nipponbare (21.00, 23.25, 24.76, and 18.55 vs. 15.65), with no difference observed in S3 (14.68; **Figure 4F**). Spikelets per panicle were significantly increased in S1 (*qSPP1*, a=22.06), S2 (*qSPP3*, a=9.78), S4 (*qSPP12-1*, a=11.22), and S5 (*qSPP12-2*, a=26.33) relative to Nipponbare (146.00, 121.00, 124.31, and 154.55 vs. 101.88), while S3 showed no significant difference (121.00; **Figure 4G**). Similarly, Grains per panicle were significantly greater in S1 (*qGPP1*, a=20.20), S2 (*qGPP3*, a=8.65), S4 (*qGPP12-1*, a=13.54), and S5 (qGPP12-2, a=27.19) than in Nipponbare (133.33, 110.24, 96.56, and 154.55 vs. 101.88), with no significant difference in S3 (121.00; **Figure 4H**). Spikelets density per 10 cm panicle was also significantly higher in S1 (*qSSD1*, a=9.64), S2 (*qSSD3*, a=5.68), S4 (*qSSD*12-1, a=6.66), and S5 (*qSSD12-2*, a=12.16) compared to Nipponbare (69.23, 61.31, 63.26, and 74.27 vs. 49.94), whereas S3 did not differ (51.29; **Figure 4I**).

Yield per plant was significantly higher in S2 (*qYD3*, a=5.04 g; 28.90 g) and S4 (*qYD12*, a=4.24 g; 27.30 g) than in Nipponbare (18.82 g), while S1, S3, and S5 (lacking yield QTL) showed no significant difference (23.10, 25.47, and 17.60 g; **Figure 4J**). Plant height was significantly increased in S1 (*qPH1*, a=5.05 cm), S4 (*qPH12-1*, a=5.55 cm), and S5 (*qPH12-2*, a=3.50 cm) relative to Nipponbare (103.00, 104.00, and 99.88 cm vs. 92.90 cm), with no difference in S2 and S3 (94.50 and 93.93 cm; **Figure 4K**). Finally, panicle length was significantly longer in S1 (*qPL1*, a=1.32 cm) and S4 (*qPL12*, a=1.82 cm) than in Nipponbare (23.00 and 24.00 cm vs. 20.37 cm), while S2, S3, and S5 (lacking panicle length QTL) did not differ significantly (19.90, 20.98, and 20.82 cm; **Figure 4L**).

### 3.6 Cytological analysis of glume cells in SSSLs (S1-S5) carrying different QTL for grain size

**Figure 5:**
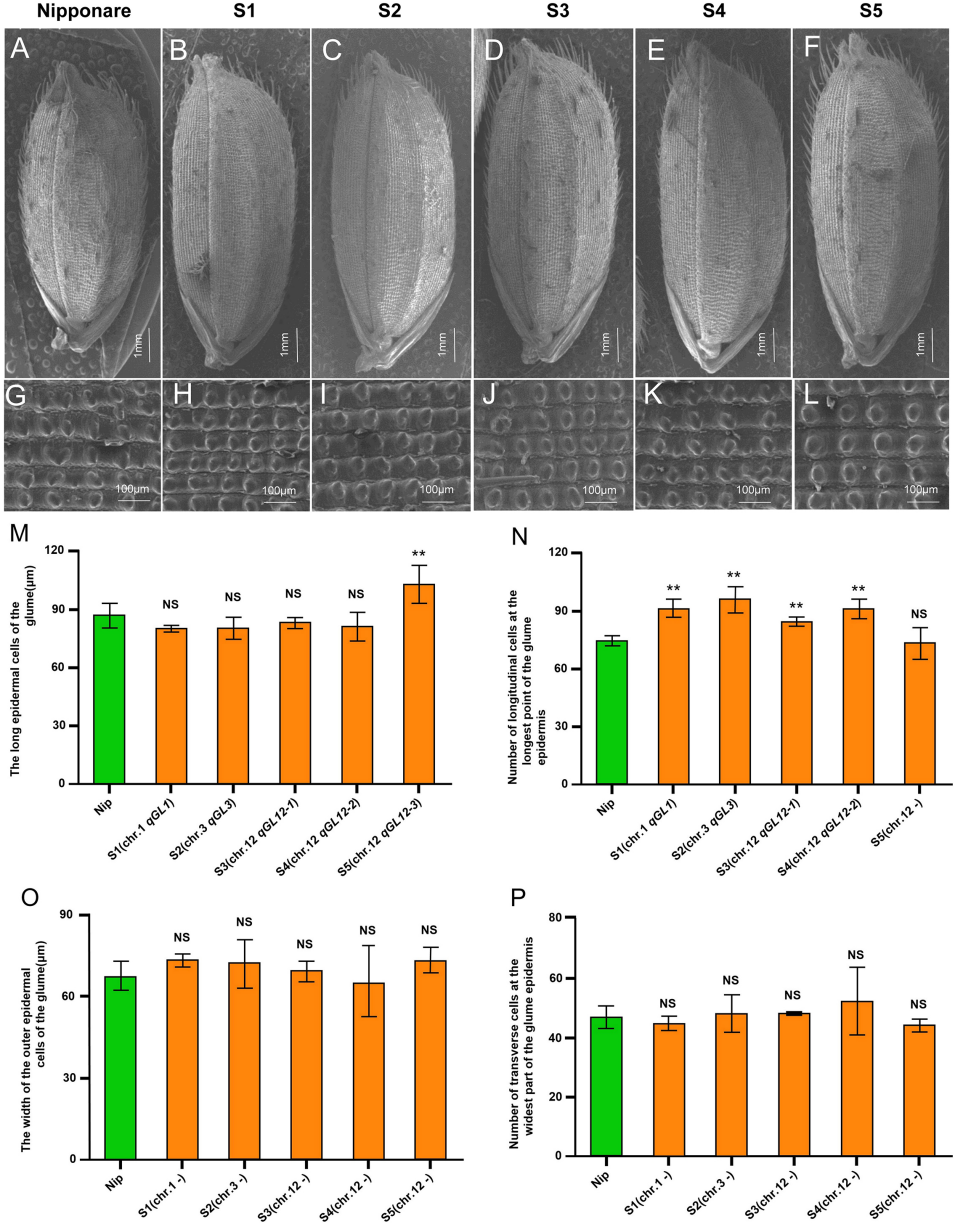
Scanning electron microscope observation and analysis of hulls of S1-S5. **(A–P)** Scanning electron microscopy images of grain **(A–E)** and outer epidermis **(G–L)**, **(M)** Average cell length of outer epidermis, **(N)** Mean longitudinal cell number of outer epidermis, **(O)** Average cell width of outer epidermis, P: Average lateral cell number of outer epidermis, *P* < 0.05 indicates significant difference between Japanese sunny and S1-S5 in the two-tailed t test.

Grain length was significantly increased in five single-segment substitution lines (SSSLs)—S1 (*qGL1*, a=0.22), S2 (*qGL3*, a=0.27), S3 (*qGL12-1*, a=0.17), S4 (*qGL12-2*, a=0.24), and S5 (*qGL12-3*, a=0.35)—compared to the recipient parent Nipponbare. None of these lines carry grain-width QTL, and their grain width did not differ significantly from Nipponbare (**Figures 5A–F**). To investigate the cytological basis of the grain-length increase conferred by these QTL, we examined glume cell morphology in the five SSSLs and Nipponbare using scanning electron microscopy (**Figures 5G–L**). The average longitudinal cell number in the lemma was significantly higher in S1–S4 (91, 95, 85, and 91, respectively) than in Nipponbare (74), corresponding to increases of 23%, 29%, 14%, and 23%. In contrast, S5 (73) showed no significant difference from Nipponbare (**Figure 5N**). Measurements of lemma epidermal cell length using Image J revealed that cell length in S5 (102.97 µm) was significantly greater—by 19%—than in Nipponbare (86.84 µm). By contrast, cell lengths in S1–S4 (80.19, 80.41, 83.26, and 80.88 µm, respectively) did not differ significantly from Nipponbare (**Figure 5M**). These results indicate that the increased grain length in S1–S4 results from a higher cell number, whereas in S5 it is due to enhanced longitudinal cell expansion. This suggests that *qGL1*, *qGL3*, *qGL12-1*, and *qGL12–2* may regulate cell division, while *qGL12–3* likely influences cell expansion. No significant differences were observed in lemma epidermal cell width among S1–S5 (73.26, 71.73, 69.27, 65.05, and 73.13 µm, respectively) and Nipponbare (67.32 µm) (**Figure 5O**). Similarly, transverse cell numbers did not differ significantly between the SSSLs (45, 48, 48, 52, and 44, respectively) and Nipponbare (47) (**Figure 5P**), indicating no transverse cell expansion in the SSSL grains relative to Nipponbare.

## 4 Discussion

### 4.1 Single segment substitution lines (S1-S5) (Derived from CSSL-Z799)are a valuable resource for molecular-designed rice breeding

With advances in molecular marker-assisted selection (MAS) breeding, Chromosome Segment Substitution Lines (CSSLs) are now widely applied in various crops such as rice, rapeseed, maize, and wheat. Through hybridization and MAS, desirable traits from different CSSLs can be pyramided into a single variety rapidly and accurately. Zhang (2021), for instance, established a library of rice SSSLs and used it to successfully design and breed four new rice varieties that met predetermined specifications.

In this study, we develop a CSSL designated Z799, which carries 10 substitution segments from the donor restorer line R225 in the genetic background of the recipient parent Nipponbare. Through QTL analysis of a Nipponbare/Z799 F_2_ population and subsequent MAS, we developed five SSSLs (S1-S5), each harboring key QTL for yield-related traits. These SSSLs not only validated 15 QTL previously detected in the F_2_ population — including *qPL1*, *qNPB1*, *qNPB12-2*, *qPH1*, *qNSB1*, *qNSB12*, *qSPP1*, *qGPP1*, *qSDD1*, *qGL1*, *qGL12-1*, *qGL3*, *qRLW3*, *qRLW12-1*, *qGWT1*—but also uncovered 18 novel QTL (*qGL12-2*, *qSPP3*, *qSPP12*, *qGPP3*, *qGPP12*, *qRLW1*, *qRLW12-2*, *qGWT3*, *qGWT12*, *qNPB3*, *qNPB12-1*, *qNSB3*, *qSDD3*, *qSDD12*, *qYD3*, *qYD12*, *qPH12*, *qPL12*). This demonstrated that SSSLs offer superior QTL detection efficiency compared to F_2_ populations, significantly improving mapping accuracy while enriching the genetic resources available for rice breeding. Phenotypic evaluation revealed significant trait improvements across the SSSLs. For example, grain length increased significantly in all five SSSLs compared to Nipponbare (7.33 mm), reaching up to 8.01 mm in S5. Similarly, ratio of grain length-to-width, thousand-grain weight, panicle branch number, spikelets per panicle, and yield per plant were notably enhanced in specific SSSLs. Detailed trait measurements for each line are summarized in **Figures 4A–L**. In conclusion, the five SSSLs derived from Z799 all harbor favorable QTL for yield-related traits. They provide a robust foundation for future fine-mapping, map-based cloning, and functional studies of these QTL, while also serving as practical breeding resources for designed high-yield breeding in *japonica* rice-growing regions.

### 4.2 Comparison of QTL carried by Z799 with previously reported genes

Through QTL analysis of the Nipponbare/Z799 F_2_ population and the subsequently developed SSSLs, we identified 33 QTL associated with yield-related traits in rice and compared them with previously reported genes in corresponding intervals.

On chromosome 1, *qGL1*, *qPL1*, *qPH1*, *qNPB1*, *qNSB1*, *qSPP1*, *qGWT1*, *qRLW1*, *qGPP1*, and *qSSD1* are all linked to the molecular marker RM8111 (6.27 Mb). Within this interval, we selected two potential candidate genes, *SDG721* and *OsFTL1*, located approximately 0.23 Mb and 0.22 Mb from this marker, respectively. The mutant *sdg721* exhibits gibberellin-deficient phenotypes compared to the wild type, including semi-dwarfism, shortened cell length, and reduced number of secondary branches and grains per panicle (Jiang et al., 2018), making it a potential candidate gene for *qNSB1* and *qGPP1*. *FT-L1* encodes a florigen-like protein that promotes flowering in rice. The transport of florigens Hd3a and RFT1 to the shoot apical meristem activates *FT-L1*. The gene enhances the effects of Hd3a and RFT1 during the transition of the vegetative meristem to the inflorescence meristem and organizes panicle branching by increasing the determinacy of distal meristems, thereby affecting primary branch number and panicle length (Giaume et al., 2023), suggesting it as a candidate for *qNPB1* and *qPL1*.

On chromosome 3, QTL (*qGL3*, *qNPB3*, *qNSB3*, *qSPP3*, *qGWT3*, *qRLW3*, *qGPP3*, *qSSD3*, *qYD3*) are linked to the molecular marker RM5928 (12.55 Mb). Three potential candidate genes—*OsSUS4*, *OsFUG1*, and *ACE1*— were screened in the region, located approximately 1.62 Mb, 0.25 Mb, and 0.35 Mb from this marker, respectively. Overexpression of *OsSUS4* significantly increased the thousand-grain weight and yield per plant in transgenic plants, with no significant differences in tiller number per plant, seeds per panicle, or seed setting rate (Fan et al., 2019), suggesting it is a potential candidate for *qGWT3* and *qYD3*. The *OsFUG1* knockout mutant *fug1* showed dwarfism, defects in fertility, grain weight, and panicle morphology (Rosa et al., 2018), indicating it as a potential candidate for *qYD3* and *qNSB3*. *ACE1* may be involved in GA signaling (Han et al., 2005). It encodes a protein of unknown function whose expression confers the capacity for cell division in the intercalary meristem zone, promoting internode elongation in the presence of gibberellin and increased grain length (Bailey-Serres and Voesenek, 2020), making it a candidate for *qGL3*.

On chromosome 12, QTL (*qGL12-1*, *qNPB12-1*, *qGWT12*, *qRLW12*) are linked to the molecular marker RM27819 (7.6 Mb). Within this interval, we identified *OsAK3* as a candidate gene. *OsAK3* encodes an adenylate kinase that regulates grain size by controlling cell growth in the spikelet hull. Compared to the wild type Dongjin (DJ), the *osak3* mutant has fewer tillers, exhibits dwarfism and shortened internodes but increased internode number, and shows significantly reduced grain length, grain width, and thousand-grain weight, along with lower sensitivity to exogenous Brassinolide (BL) treatment. Overexpression of *OsAK3* increased grain length. Cytological observation revealed that lemma cell length and width were significantly reduced in *osak3*, while cell number increased, indicating that *OsAK3* modulates grain size by regulating cell growth (Zhang et al., 2021), supporting its candidacy for *qGL12-1*.

In another interval on the same chromosome, QTL (*qGL12-2*, *qPL12*, *qPH12*, *qNPB12-2*, *qNSB12*, *qSPP12*, *qRLW12-2*, *qGPP12*, *qSSD12*, *qYD12*) are linked to the molecular marker RM519 (20.0 Mb). Two candidate genes, *qTGW12a* and *GNP12*, were identified approximately 0.35 Mb and 1.23 Mb from this marker, respectively. Knockout of *qTGW12a* (*LOC_Os12g36660*) in ZH11 resulted in significantly narrower grains and reduced grain weight compared to the wild type (Du et al., 2021), suggesting it as a potential candidate for *qYD12*. *GNP12* positively regulates panicle development in rice by modulating Cytokinin levels. Compared to the wild type Ce253, panicle length, grain number per panicle, grain length, and seed setting rate were decreased in the relevant materials (Pan et al., 2022), indicating its potential role in *qPL12*, *qGL12-2*, *qSSD12*, *qGPP12*, and *qSPP12*.

The limitation of the work is lack of assured candidate gene for each QTL. Future work will focus on fine-mapping to narrow down the identified QTL intervals, refining the candidate gene list, and combining sequencing of relevant candidates with complementation tests to validate the accuracy of the inferred candidate genes.

## Data Availability

The raw data supporting the conclusions of this article will be made available by the authors, without undue reservation.
